# Tuning lipid nanoparticle pKa to exploit the vitronectin corona for tumor-selective mRNA delivery to the lung

**DOI:** 10.1016/j.omtn.2026.103020

**Published:** 2026-08-01

**Authors:** Yoon Tae Goo, Olena R. Taratula, Oleh Taratula

**Affiliations:** 1 Department of Pharmaceutical Sciences, College of Pharmacy, Oregon State University, 2730 SW Moody Avenue, Portland, OR 97201, USA; 2 College of Pharmacy, CHA University, 335 Pangyo-ro, Bundang-gu, Seongnam-si, Gyeonggi-do 13488, Republic of Korea

## Main text

**Figure 1:**
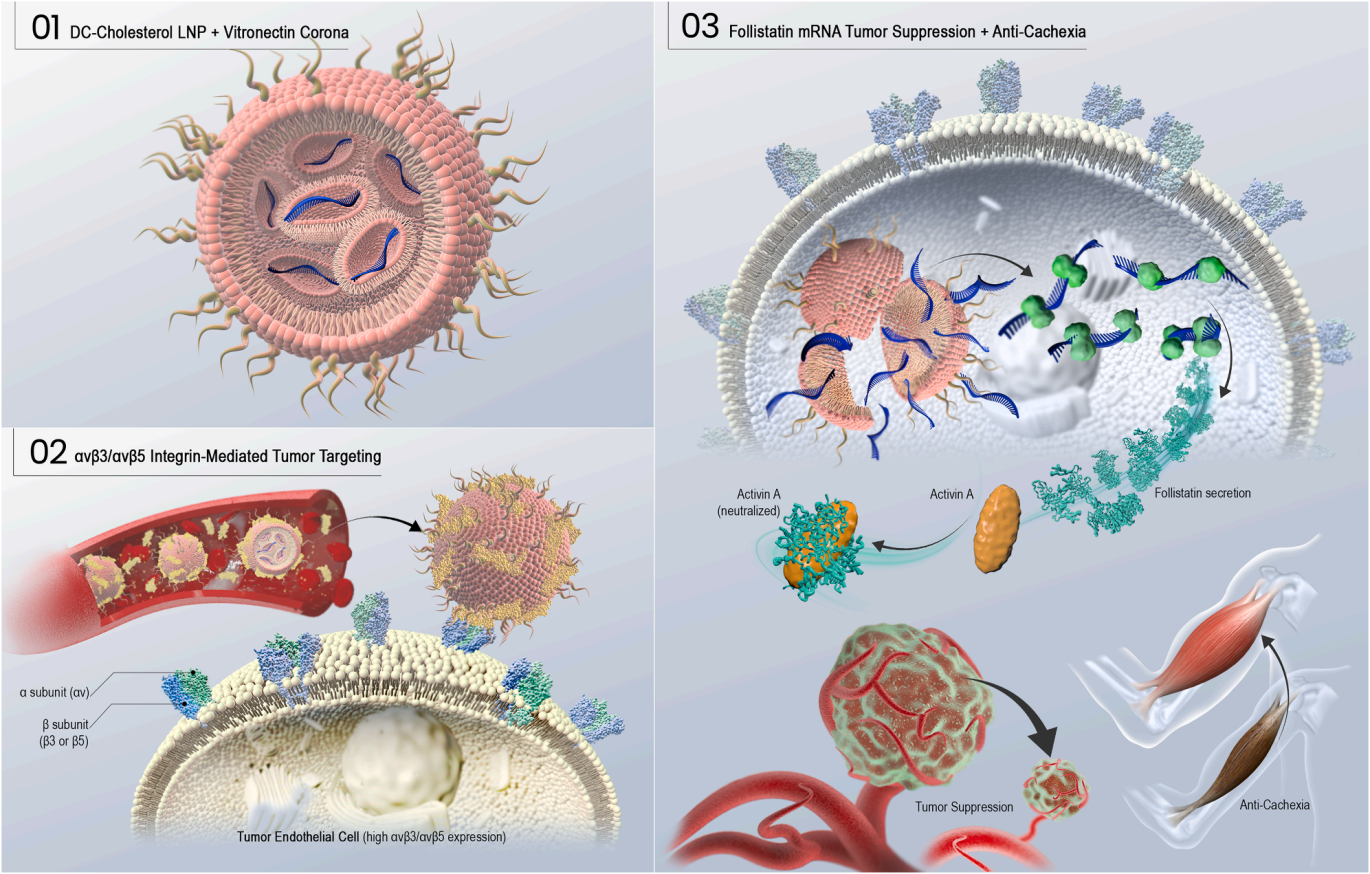
Ligand-free, formulation-level strategy for tumor-selective mRNA delivery to the lung tumor Replacing conventional cholesterol with cationic DC-cholesterol in a 113-O12B-based LNP raises the apparent pK_a_, driving the formation of a Vtn-rich protein corona. The Vtn corona acts as an endogenous ligand for α_v_β_3_/α_v_β_5_ integrins, which are highly expressed on the tumor endothelium, yielding tumor-preferential uptake. The encoded FST is secreted and neutralizes ActA, simultaneously suppressing tumor growth and reversing cachexia.

Lipid nanoparticles (LNPs) made mRNA medicines a clinical reality, but a stubborn problem has limited their reach in oncology: after intravenous injection, conventional LNPs accumulate overwhelmingly in the liver.^1^ Redirecting them to extrahepatic tumors has typically required chemically conjugating targeting ligands to the particle surface, an approach that adds manufacturing complexity and regulatory burden.^2^^,^^3^^,^^4^ In our study, recently published in the *Journal of Controlled Release*, we asked whether a single, formulation-level change to commercially available components could instead teach an LNP to deliver mRNA not merely to a specific organ, but preferentially to a tumor growing within that organ (Figure 1). We found that it could.

### A one-component swap reprograms biodistribution

Our starting point was an LNP built around the commercially available ionizable lipid 113-O12B. By replacing the conventional cholesterol in this formulation with cationic DC-cholesterol and changing nothing else, we generated a particle we call DC_113 that, after systemic administration, expressed its mRNA cargo exclusively in the lungs. Whole-body and *ex vivo* bioluminescence imaging of luciferase mRNA confirmed that the parent cholesterol formulation was localized to the spleen and liver, whereas DC_113 produced a clean, lung-restricted signal. Fluorescent-lipid tracking established that this reflected genuine particle accumulation in the lung rather than expression alone.

### Vitronectin in the protein corona is the targeting switch

We traced this lung tropism to the protein corona that forms on the particle in blood. DC_113 strongly recruited plasma vitronectin (Vtn), a ligand for the αvβ3 and αvβ5 integrins that are abundant in lung vasculature.^5^ The parent formulation bound essentially no Vtn. To understand why, we built and screened a systematic library of 20 formulations—10 ionizable lipids, each paired with conventional or DC-cholesterol—and uncovered a clean, quantitative rule: the apparent pK_a_ of the assembled LNP governs Vtn capture and, in turn, lung delivery. Exclusive lung targeting emerged only when DC-cholesterol pushed the apparent pK_a_ above ∼7.9, a value reached only by triamine ionizable lipids. Monoamine and diamine lipids did not reach this threshold, even in the presence of DC-cholesterol and, therefore, were delivered predominantly to the liver and spleen, with only partial accumulation in the lungs. Molecular docking and molecular dynamics simulations provided a mechanistic explanation for this trend. Vitronectin (Vtn) binding affinity increased with the number of protonatable tertiary amines in the ionizable lipid (monoamine < diamine < triamine), while DC-cholesterol further enhanced the interaction by forming an additional salt bridge that stabilized the lipid-Vtn interface.

### From organ targeting to tumor preference

The conceptual leap in our work is that this endogenous mechanism discriminates within the lungs. Because αvβ3/αvβ5 integrins are further upregulated during malignant transformation,^6^^,^^7^ the same Vtn-driven recognition that brings DC_113 to the lung also concentrates it in the tumor tissue. In an orthotopic A549 lung cancer model, we measured a 5.8-fold increase in integrin αv in tumor versus adjacent lung, and DC_113 delivered 3.2-fold more mRNA to the tumor than to healthy lungs.

### Local production of follistatin outperforms systemic delivery

To test therapeutic consequence, we loaded DC_113 with mRNA encoding follistatin (FST), an endogenous antagonist of activin A (ActA),^8^ a driver of both tumor progression and cancer cachexia.^9^^,^^10^ Strikingly, DC_113 achieved roughly 2.5-fold greater tumor suppression than liver-tropic LNPs, despite those liver-tropic particles generating higher circulating FST. The advantage came from spatial control: producing FST at the tumor neutralized ActA at its source before it reached circulation. Beyond shrinking tumor burden, the therapy reversed cachexia, preserving food intake, body weight, muscle mass, and adipose depots, while downregulating the muscle-atrophy genes *Foxo1*, *Fbxo32*, and *Murf1*. Repeat dosing showed no hematologic or biochemical toxicity.

### Why this matters

Our findings reframe protein-corona engineering from a tool for organ selection into one capable of intratumoral preference, achieved with a ligand-free, single-component substitution of off-the-shelf materials. The pK_a_-to-Vtn-binding relationship we define is a predictive design rule that others can apply to tune LNPs toward the lung, and the broad overexpression of αv integrins and ActA across lung cancer subtypes suggests that the platform may generalize beyond the adenocarcinoma model studied here. By coupling a manufacturable delivery strategy with a payload that simultaneously addresses a primary tumor and its systemic wasting syndrome, DC_113 offers a translationally minded blueprint for mRNA-based cancer therapy.

## Acknowledgments

This work was supported by the National Cancer Institute of the National Institutes of Health (R01CA237569 and R37CA234006) and the Eunice Kennedy Shriver National Institute of Child Health and Human Development (R01HD101450 and R01HD112007). Additional support was provided by the Basic Science Research Program through the National Research Foundation of Korea (NRF), funded by the Ministry of Education (RS-2023-00241580).

## Declaration of interests

O.T., O.R.T., and Y.T.G. are named inventors on a US patent application (attorney docket no. 245-114811-01/OSU-25-38), “Lipid Nanoparticles for Targeted mRNA Delivery and Methods of Using the Same,” filed by Oregon State University on September 18, 2025, which relates to the subject matter of this work.
